# Role of vitamin C infusion in postoperative mechanically ventilated neonates with sepsis: a randomized controlled trial

**DOI:** 10.1007/s00431-025-06625-4

**Published:** 2025-11-29

**Authors:** Asmaa Mahmoud Elmesiry, Mai Rabie Elsheikh, Khalid Mohamed Elshimy, Amany Mohamed Abotaleb

**Affiliations:** 1 Pediatrics and Neonatology Department, Faculty of Medicine, Tanta University, Alestad Street, Tanta, 31527 Al Gharbia Egypt; 2 Pediatric Surgery Unit, General Surgery Department, Faculty of Medicine, Tanta University, Tanta, Egypt; 3 Surgical Intensive Care, and Pain Medicine Department, Faculty of Medicine, Tanta University, Tanta, Egypt

**Keywords:** Neonatal sepsis, Vitamin C, Mechanical ventilation, Inotrope, Respiratory parameters

## Abstract

This research investigated the efficacy of intravenous vitamin C administration in septic, mechanically ventilated (MV) full-term neonates following surgical interventions. This double-blinded randomized controlled trial included 50 full-term neonates who required mechanical ventilation and developed confirmed sepsis after surgery. Neonates were randomly assigned to receive either standard sepsis management with placebo (No Vitamin C group) or standard protocol with vitamin C infusion, administered as a 0.5 g/kg loading dose followed by a maintenance dose of 0.5 g/kg/h over 6 h, continued for 7 to 10 days (Vitamin C group). Respiratory rate and peak inspiratory pressure were significantly lower at 24 h, 72 h, and 120 h in the Vitamin C group than in the No Vitamin C group. FiO₂ requirements were significantly reduced at 72 h and 120 h in the Vitamin C group. SpO_2_/FiO_2_ did not change across groups at baseline and 24 h but were considerably higher in the Vitamin C group at 72 h and 120 h. Duration of MV (4.44 ± 1.23 vs. 5.64 ± 2.2 days, *p* = 0.021) and inotropic support needs (40% vs. 76%, *p* = 0.010) were significantly lower in the Vitamin C group. There were no statistically significant differences in mortality rates or the duration of hospitalization, including stays at neonatal intensive care units (NICU) or the hospital.

*Conclusion*: Vitamin C infusion significantly improved respiratory parameters and reduced the duration of MV and inotropic support requirements in septic neonates following surgery, though it did not significantly affect the NICU or hospital length of stay or mortality.

*Trial registration*: registered on ClinicalTrials.gov (ID: NCT06780345) (date: 17/1/2025).

**Table:** 

**What is Known:** • *In neonatal intensive care units (NICU), neonatal sepsis continues to be a major cause of death and morbidity. * • *Vitamin C has become a possible therapeutic intervention given its many pathways in sepsis control. *
**What is New:** • *Vitamin C infusion significantly improved respiratory parameters and reduced the duration of MV and inotropic support requirements in septic neonates following surgery, though it did not significantly affect the NICU or hospital length of stay or mortality. *

**What is Known:**

• *In neonatal intensive care units (NICU), neonatal sepsis continues to be a major cause of death and morbidity.
*

• *Vitamin C has become a possible therapeutic intervention given its many pathways in sepsis control.
*

**What is New:**

• *Vitamin C infusion significantly improved respiratory parameters and reduced the duration of MV and inotropic support requirements in septic neonates following surgery, though it did not significantly affect the NICU or hospital length of stay or mortality.
*

## Introduction

Globally, neonatal sepsis is a major health issue, characterized by systemic infection with bacteremia within 28 days after birth [1]. Still, a key factor of death and morbidity in neonatal intensive care units (NICU) all over is this illness [2, 3].

The surgical neonates are more prone to infection due to invasive procedures and contact with pathogenic bacteria in the hospital environment. Availability of better antibiotics has reduced deaths due to infection, but sepsis is still a significant cause of death among newborn surgical patients [4].

While antibiotic intervention is essential, it presents its own challenges, potentially leading to adverse outcomes in uninfected infants [5–7]. This has prompted an investigation into novel diagnostic approaches and therapeutic strategies [8]. Vitamin supplementation, particularly vitamin C, has garnered significant attention among these emerging interventions. Vitamin C, an essential micronutrient, demonstrates promising potential in sepsis management through its diverse biological functions [9].

Recent research has revealed that critically ill septic patients typically present with depleted vitamin C levels, corresponding with rising organ malfunction and death [10]. The therapeutic potential of vitamin C in sepsis is attributed to its multifaceted mechanisms, including its role as an enzymatic cofactor in catecholamine and cortisol production [11], its antioxidant properties in managing reactive oxygen species [12], and its ability to preserve capillary blood flow and vasoactive medication–induced vascular responsiveness [13].

This study aims to evaluate the effectiveness of intravenous (IV) vitamin C for septic neonates, mechanically ventilated (MV) full-term neonates following surgical interventions, specifically examining its impact on ventilation parameters, weaning time, and inotropic support requirements.

Patients and methods.

This double-blinded randomized controlled trial included 50 full-term neonates (gestational age > 37 weeks) who required MV and developed clinically and serologically confirmed sepsis after undergoing surgical procedures. The study was conducted in the NICU at Tanta University Hospital, Egypt, from February 2023 to August 2023. The trial was approved by the institutional ethics committee (approval ID: 36264PR117/2/23), was done in accordance with the Declaration of Helsinki, and was registered on ClinicalTrials.gov (ID: NCT06780345) (date: 17/1/2025). Written informed consent was obtained from all participating neonates’ parents or legal guardians.

Neonates were excluded from the study if they presented with major congenital anomalies or chromosomal abnormalities or were born at a gestational age of less than 37 weeks. Additional exclusion criteria included the presence of hypoxic ischemic encephalopathy, neuromuscular diseases, or intraventricular hemorrhage.

### Randomization and blindness

Using computer-generated random numbers, neonates were divided into two equal groups (using https://www.randomizer.org/) in sealed opaque envelopes. No Vitamin C group (control group), who received the standard sepsis management protocol with a placebo, and Vitamin C group, who received the standard protocol along with a vitamin C infusion, administered as a 0.5 g/kg loading dose followed by a maintenance dose of 0.5 g/kg/h over 6 h, continued for 7 to 10 days [14].

The attending NICU physicians responsible for data collection and the parents or guardians of the neonates were blinded to the group allocation and the treatment administered.

All enrolled neonates underwent a comprehensive evaluation, including history-taking and a thorough clinical examination. Laboratory investigations comprised a complete blood count, liver function tests, blood urea nitrogen, creatinine levels, arterial blood gas analysis, and C-reactive protein measurement.

Study parameters, including mean arterial blood pressure (MAP), heart rate (HR), and arterial oxygen saturation (SpO₂), were continuously monitored in both groups. Mechanical ventilation parameters, including respiratory rate (RR), peak inspiratory pressure (PIP), and fraction of inspired oxygen (FiO₂), were recorded at baseline, 24 h, 72 h, and 120 h following the initiation of the infusion.

The need for inotropic support was documented. The total duration of MV was recorded at discharge, along with the length of hospital stay and mortality outcomes.

The primary outcome was the duration of MV. Secondary outcomes included the need for inotropic support and mortality rates.

### Sample size calculation

G*Power 3.1.9.2 (Universität Kiel, Germany) was used to calculate the sample size. A pilot study, including five cases in each group, revealed that the mean (± standard deviation) duration of MV was 6.6 ± 1.95 days in the study group and 8.6 ± 1.52 days in the control group. The sample size determination was based on an effect size of 1.14, a 95% confidence level, and a study power of 95%, with an equal allocation ratio (1:1) between groups. Additionally, four participants were added to each group to account for potential dropouts. Consequently, a total of 25 patients will be recruited per group.

### Statistical analysis

We used SPSS version 27 (IBM©, Armonk, NY, USA) for statistical analysis. The Shapiro–Wilk test and histograms were used to determine whether the data distribution was normal. Mean and standard deviation (SD) were used to present quantitative parametric data, which were examined using the unpaired Student’s *t*-test. For qualitative variables, frequency and percentage were used for description, and the chi-square or Fisher’s exact test was used for evaluation, as appropriate. Statistical significance was determined by a two-tailed *p* value less than 0.05.

## Results

**Fig. 1:**
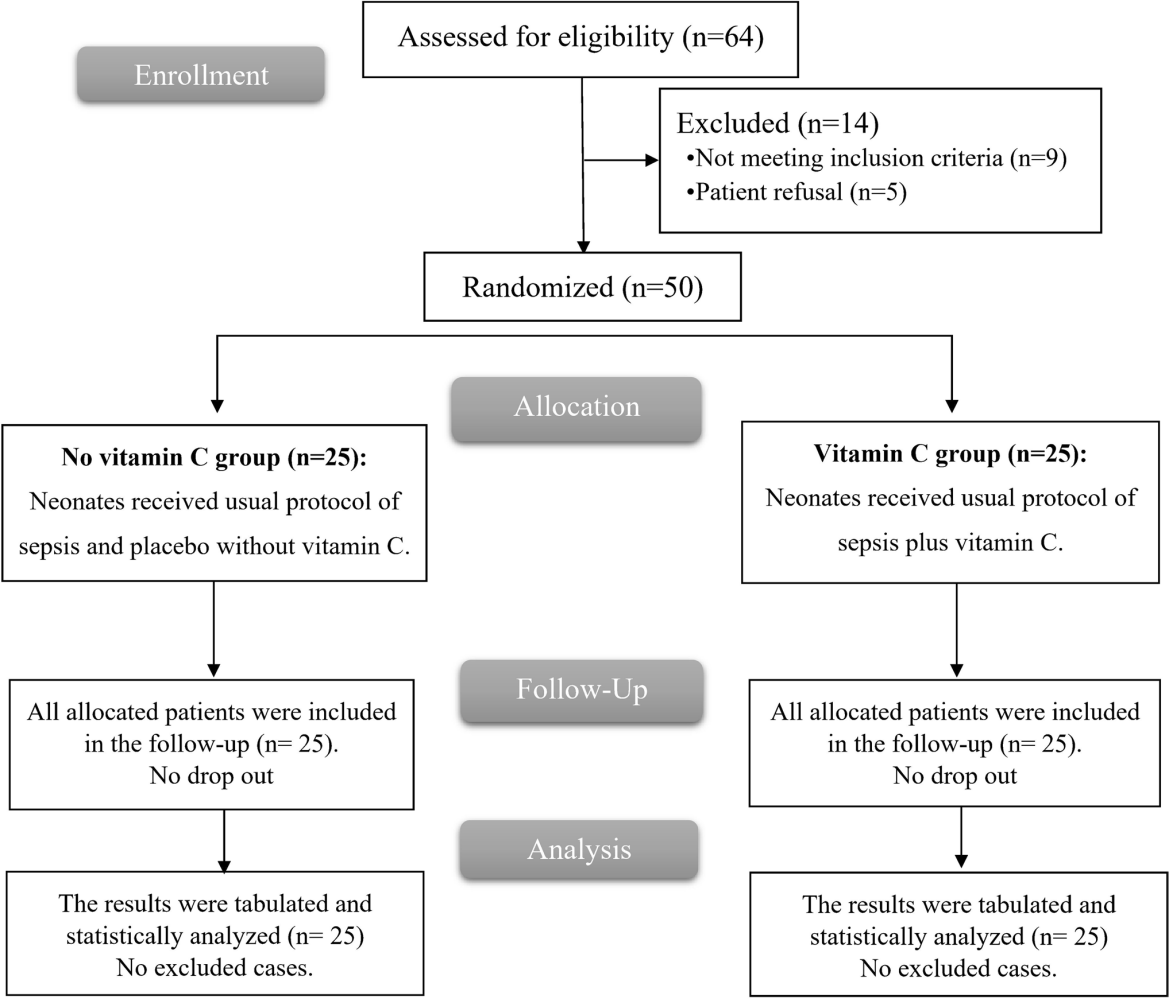
CONSORT flowchart of the enrolled patients

Of the 64 neonates originally screened for this research, nine were removed because they did not meet eligibility requirements, and five were removed due to parental rejection. The remaining neonates were randomly allocated into two groups of 25 neonates. Every neonate in these groups was observed and statistically analyzed throughout the trial (Fig. 1).

Both groups had similar birth weight, sex, gestational age, delivery method, % of prenatal steroids, and SNAPPE-II score (Table 1).

**Table 1:** Demographic data of the studied groups

		No Vitamin C group (*n* = 25)	Vitamin C group (*n* = 25)	*p* value	Mean difference/RR (95% CI)
Postnatal age (days)		9.16 ± 1.62	8.84 ± 1.28	0.443	0.32 (− 0.51:1.15)
Gestational age (weeks)		38.56 ± 1.29	39.12 ± 1.17	0.114	− 0.56 (− 1.26:0.14)
Birth weight (g)		1930.8 ± 191.15	1875.52 ± 130.35	0.238	55.28 (− 37.76:148.32)
Sex	Male	14 (56%)	12 (48%)	0.571	1.17 (0.68:1.99)
	Female	11 (44%)	13 (52%)		
Mode of delivery	Vaginal delivery	6 (24%)	9 (36%)	0.355	0.67 (0.28:1.59)
	Cesarean section	19 (76%)	16 (64%)		
% of prenatal steroids		14 (56%)	16 (64%)	0.564	(0.56:1.38)0.88
SNAPPE-II score		43.08 ± 6.23	45.12 ± 7.28	0.293	− 2.04 (− 5.89:1.81)

Data expressed as mean ± SD or frequency (%). *RR* relative risk, *CI* confidence interval

HR, MAP, and oxygen saturation were insignificantly different at baseline, 24 h, 72 h, and 120 h between the two groups (Table 2).

**Table 2:** Heart rate, mean arterial blood pressure, and oxygen saturation of the studied groups

	No Vitamin C group (*n* = 25)	Vitamin C group (*n* = 25)	*p* value	Mean difference (95% CI)
Heart rate (beats/min)				
Baseline	164.32 ± 8.04	164.8 ± 11.16	0.862	− 0.48 (− 6.01:5.05)
24 h	159.16 ± 7.8	154.68 ± 11.68	0.117	4.48 (− 1.17:10.13)
72 h	151.43 ± 10.38	147.36 ± 13.48	0.250	4.07 (− 2.77:10.92)
120 h	144.38 ± 8.9	140 ± 11.87	0.173	4.38 (− 1.59:10.35)
Mean arterial blood pressure (mmHg)				
Baseline	55.24 ± 6.59	56.6 ± 6.34	0.461	− 1.36 (− 5.04:2.32)
24 h	58.8 ± 7.59	60.72 ± 7.72	0.380	− 1.92 (− 6.28:2.44)
72 h	61.96 ± 8.77	63.92 ± 7.86	0.417	− 1.96 (− 6.7:2.77)
120 h	65.67 ± 9.54	66.13 ± 6.25	0.848	− 0.46 (− 5.05:4.13)
Oxygen saturation (%)				
Baseline	90.68 ± 1.46	90.92 ± 0.76	0.470	− 0.24 (− 0.9:0.42)
24 h	95.76 ± 3.03	96.28 ± 1.88	0.470	− 0.52 (− 1.95:0.91)
72 h	96.83 ± 2.76	97.32 ± 2.06	0.483	− 0.49 (− 1.88:0.89)
120 h	97.95 ± 0.74	98.04 ± 1	0.738	− 0.09 (− 0.59:0.41)

Data expressed as mean ± SD. *CI* confidence interval

The Vitamin C group had considerably reduced respiratory rate (*p* = 0.006, 0.004, and 0.002, respectively) and peak inspiratory pressure (*p* = 0.007, 0.026, and 0.039, respectively) at 24 h, 72 h, and 120 h compared to the No Vitamin C group. FiO_2_ levels did not change across groups at baseline and 24 h but were considerably lower in the Vitamin C group at 72 h and 120 h (*p* = 0.047 and 0.023, respectively). SpO_2_/FiO_2_ did not change across groups at baseline and 24 h but were considerably higher in the Vitamin C group at 72 h and 120 h (*p* = 0.020 and 0.022, respectively) (Table 3).

**Table 3:** Respiratory rate, peak inspiratory pressure, and FiO_2_ of the studied groups

	**No Vitamin C group (*n* = 25)**	**Vitamin C group (*n* = 25)**	***p* value**	**Mean difference (95% CI)**
Respiratory rate (breaths/min)				
Baseline	51.24 ± 3.69	49.84 ± 3.44	0.171	1.4 (− 0.63:3.43)
24 h	49.28 ± 3.79	46.6 ± 2.69	0.006*	2.68 (0.81:4.55)
72 h	42.57 ± 3.81	39.28 ± 3.71	0.004*	3.29 (1.15:5.43)
120 h	36.86 ± 3.45	33.63 ± 3.25	0.002*	3.23 (1.32:5.14)
Peak inspiratory pressure (cm H_2_O)				
Baseline	20.28 ± 3.42	19.16 ± 2.84	0.214	1.12 (− 0.67:2.91)
24 h	17.4 ± 4.18	14.32 ± 3.48	0.007*	3.08 (0.89:5.27)
72 h	13.04 ± 4.2	10.72 ± 2.67	0.026*	2.32 (0.32:4.33)
120 h	9.14 ± 1.61	7.45 ± 2.25	0.039*	1.69 (0.57:2.8)
FiO_2_ (%)				
Baseline	63.6 ± 15.24	62.8 ± 15.68	0.856	0.8 (− 7.99:9.59)
24 h	52 ± 17.56	47.2 ± 14.29	0.294	4.8 (− 4.31:13.91)
72 h	42.17 ± 15.65	34.4 ± 10.44	0.047*	7.77 (0.21:15.34)
120 h	33.81 ± 6.69	30.42 ± 2.04	0.023*	3.39 (0.58:6.21)
SpO_2_/FiO_2_				
Baseline	1.51 ± 0.38	1.54 ± 0.41	0.765	− 0.03 (− 0.26:0.19)
24 h	2.06 ± 0.7	2.23 ± 0.67	0.382	− 0.17 (− 0.56:0.22)
72 h	2.54 ± 0.72	2.97 ± 0.52	0.020*	− 0.43 (− 0.79: − 0.08)
120 h	2.99 ± 0.48	3.23 ± 0.17	0.022*	− 0.25 (− 0.45: − 0.04)

Data expressed as mean ± SD. *CI* confidence interval, *FiO*_*2*_ fraction of inspired oxygen

* Statistically significant at *p* ≤ 0.05

MV duration and inotropic requirement were considerably reduced in the Vitamin C group compared to the No Vitamin C group (*p* = 0.021 and 0.010, respectively). Insignificant differences were seen in ventilator-free days, NICU and hospital stays, and mortality (Table 4).

**Table 4:** Duration of MV, inotropic need, ventilator-free days, length of NICU and hospital stay, and mortality of the studied groups

	**No Vitamin C group (*n* = 25)**	**Vitamin C group (*n* = 25)**	***p* value**	**Mean/median difference/RR (95% CI)**
Duration of MV (days)	5.64 ± 2.2	4.44 ± 1.23	0.021*	1.2 (0.19:2.21)
Inotropic need	19 (76%)	10 (40%)	0.010*	1.9 (1.12:3.22)
Ventilator-free days	3.32 ± 2.27	3.72 ± 1.9	0.503	− 0.4 (− 1.59:0.79)
Length of NICU stay (days)	9 (7–12)	8(7–9)	0.338	− 1 (− 3:1)
Length of hospital stay (days)	11 (9–14)	11(10–12)	0.792	0 (− 3:2)
Mortality	6 (24%)	3 (12%)	0.463	2 (0.56:7.12)

Data expressed as mean ± SD or frequency (%). *RR* relative risk, *CI* confidence interval, *MV* mechanical ventilation, *NICU* neonatal intensive care unit

* Statistically significant at *p* ≤ 0.05

## Discussion

Vitamin C plays a role in sepsis by preserving endothelial function and improving microcirculatory flow [15, 16]. Our findings contribute significantly to understanding vitamin C’s role in managing postoperative sepsis in MV neonates.

Our study demonstrated a significant reduction in MV duration in the Vitamin C group compared to No Vitamin C group. This finding aligns with El Driny et al. [17], who reported improved outcomes in MV septic patients receiving high-dose vitamin C infusion.

The marked reduction in inotropic support requirements in our Vitamin C group parallels the findings of Zabet et al. [18], who observed significantly lower norepinephrine doses and reduced vasopressor support duration in adult septic shock patients taking vitamin C.

However, our results contrast with those of some adult studies. For instance, Lamontagne et al. [19], in their large-scale trial, found no improvement in organ dysfunction scores or vasopressor requirements with vitamin C administration. Similarly, Ahn et al. [20] reported no difference in shock reversal time between the vitamin C and control groups. This disparity might be attributed to the unique physiological characteristics of neonates and the specific context of postoperative sepsis.

However, our analysis indicated a trend toward lower mortality rates and shorter NICU and hospital stays in the vitamin C group; these variations did not approach statistical significance. This contrasts with Muhammad et al. [21]; their meta-analysis of septic patients noted a statistically significant drop in mortality and sequential organ failure assessment (SOFA) scores.

The significant improvement in respiratory parameters observed in our vitamin C group, particularly the reduced respiratory rates and peak inspiratory pressure requirements from 24 h onward, represents a notable finding. The marked reduction in FiO₂ requirements at 72 h and 120 h suggests enhanced pulmonary function and oxygen utilization. These findings can be understood through the mechanism proposed by Kashiouris et al. [22], who described vitamin C’s role in reducing oxidative stress and enhancing endovascular integrity, which may explain its contribution to improved pulmonary function, shorter ventilation duration, and greater hemodynamic stability through its anti-inflammatory effects, vasopressor synthesis support, and endothelial function enhancement.

The physiological basis for these improvements may be explained by Anand et al. [23] discussion of vitamin C’s antioxidant properties and its role in modulating inflammatory response. Our findings extend the understanding of these mechanisms in neonates, where the impact appears more pronounced than in adult populations. The recent study by Mangshetty et al. [24] supports our findings, demonstrating improved outcomes in neonatal sepsis with vitamin C supplementation, including reduced NICU stay duration (7.73 vs. 10.91 days, *p* = 0.003).

Our study showed gradual HR, MAP, and oxygen saturation improvements from baseline to 120 h in both groups. Our outcomes partially match Brown et al.’s [25] meta-analysis, which found no significant differences in several hemodynamic parameters. However, our results should be interpreted in the context of Fowler et al. [26] findings, which demonstrated that vitamin C supplementation led to rapid reductions in SOFA scores and proinflammatory biomarkers, even when immediate hemodynamic differences were not apparent.

The absence of significant between-group differences in hemodynamic parameters might reflect the complex nature of sepsis pathophysiology in neonates. Muhammad et al.’s [21] systematic review suggests that while vitamin C may not dramatically alter immediate hemodynamic parameters, it can still contribute to improved overall outcomes, as evidenced by their finding of reduced mortality (OR = 0.778, 95% CI 0.635–0.954, *p* = 0.016) and SOFA scores.

This research had some limitations, including the small sample, which may limit generalizability. The single-center design restricts application to different healthcare settings. The study focused only on full-term neonates, excluding preterm infants who might respond differently to vitamin C therapy.

## Conclusions

IV vitamin C supplementation in postoperative septic full-term neonates demonstrated significant improvements in respiratory parameters, with decreased respiratory rate, peak inspiratory pressure, and FiO₂ requirements. The Vitamin C group also showed reduced MV duration and decreased need for inotropic support. However, no significant differences were observed in hemodynamic parameters, NICU or hospital stay length, or mortality rates. These findings suggest that vitamin C may be a beneficial adjunctive therapy for improving respiratory function in postoperative neonatal sepsis, though larger multi-center trials are needed.

## Data Availability

Data are available after request from corresponding author.

## Abbreviations

SpO₂: Arterial oxygen saturation
FiO₂: Fraction of inspired oxygen
HR: Heart rate
IV: Intravenous
MAP: Mean arterial blood pressure
MV: Mechanically ventilated
NICU: Neonatal intensive care units
PIP: Peak inspiratory pressure
RR: Respiratory rate
SD: Standard deviation
